# Telomere Length and the Transition to Family Caregiving in the REGARDS Study

**DOI:** 10.1093/geroni/igab046.2988

**Published:** 2021-12-17

**Authors:** William Haley, Nicole Armstrong, Ryan Irvin, Marcela Blinka, Rasika Mathias, Jeremy Walston, David Roth

**Affiliations:** 1 University of South Florida, Tampa, Florida, United States; 2 University of Alabama at Birmingham, University of Alabama at Birmingham, Alabama, United States; 3 University of Alabama at Birmingham, Birmingham, Alabama, United States; 4 Johns Hopkins University, Baltimore, Maryland, United States; 5 Johns Hopkins University, Johns Hopkins University, Maryland, United States; 6 Johns Hopkins University School of Medicine, Baltimore, Maryland, United States

## Abstract

An increase in life expectancy and an aging population has resulted in increased risks and prevalence of age-related diseases. Previous studies have shown that factors, such as chronic stress, are associated with shorter telomere length. When telomeres become critically short, cells enter a state of senescence, which is a hallmark of aging. Several prior studies examining the relationship between caregiving and telomere length have reported mixed results. The present study utilized data from the Caregiving Transitions Study, an ancillary study to the Reasons for Geographic and Racial Differences in Stroke (REGARDS) study. The difference in telomere length across an average ~8.6 years was compared between 235 incident caregivers and 229 controls. Telomere length was determined using the qPCR telomere-to-single copy gene (IFNB1) ratio (T/S) for each participant at both baseline and follow-up timepoints. Regression models controlling for age, sex, race, and baseline telomere length examined the association between caregiving status (exposure) and the telomere length change (□T/S). Sensitivity models adjusted for potential lifestyle and socioeconomic factors, including income, education, BMI, cigarette smoking, and alcohol use. We did not observe a significant association between □T/S and caregiving (beta=0.041, p=0.615). Adding lifestyle and socioeconomic factors did not change the null relationship (beta=0.062, p=0.455). In conclusion, this study provides evidence against an association between caregiving and the change in telomere length. Ultimately, more research to address the complex relationship between caregiving and telomere attrition is needed in order to prevent or reduce adverse outcomes and improve the well-being of caregivers and care recipients.

